# Integrative Computational Modeling of the Lymph Node Stromal Cell Landscape

**DOI:** 10.3389/fimmu.2018.02428

**Published:** 2018-10-23

**Authors:** Mario Novkovic, Lucas Onder, Hung-Wei Cheng, Gennady Bocharov, Burkhard Ludewig

**Affiliations:** ^1^Institute of Immunobiology, Kantonsspital St. Gallen, St. Gallen, Switzerland; ^2^Marchuk Institute of Numerical Mathematics, Russian Academy of Sciences, Moscow, Russia

**Keywords:** lymph node, stromal cells, systems biology, network topology, morphology, lymph flow, fibroblastic reticular cells, computational models

## Abstract

Adaptive immune responses develop in secondary lymphoid organs such as lymph nodes (LNs) in a well-coordinated series of interactions between migrating immune cells and resident stromal cells. Although many processes that occur in LNs are well understood from an immunological point of view, our understanding of the fundamental organization and mechanisms that drive these processes is still incomplete. The aim of systems biology approaches is to unravel the complexity of biological systems and describe emergent properties that arise from interactions between individual constituents of the system. The immune system is greater than the sum of its parts, as is the case with any sufficiently complex system. Here, we review recent work and developments of computational LN models with focus on the structure and organization of the stromal cells. We explore various mathematical studies of intranodal T cell motility and migration, their interactions with the LN-resident stromal cells, and computational models of functional chemokine gradient fields and lymph flow dynamics. Lastly, we discuss briefly the importance of hybrid and multi-scale modeling approaches in immunology and the technical challenges involved.

## Introduction

**Figure 1:**
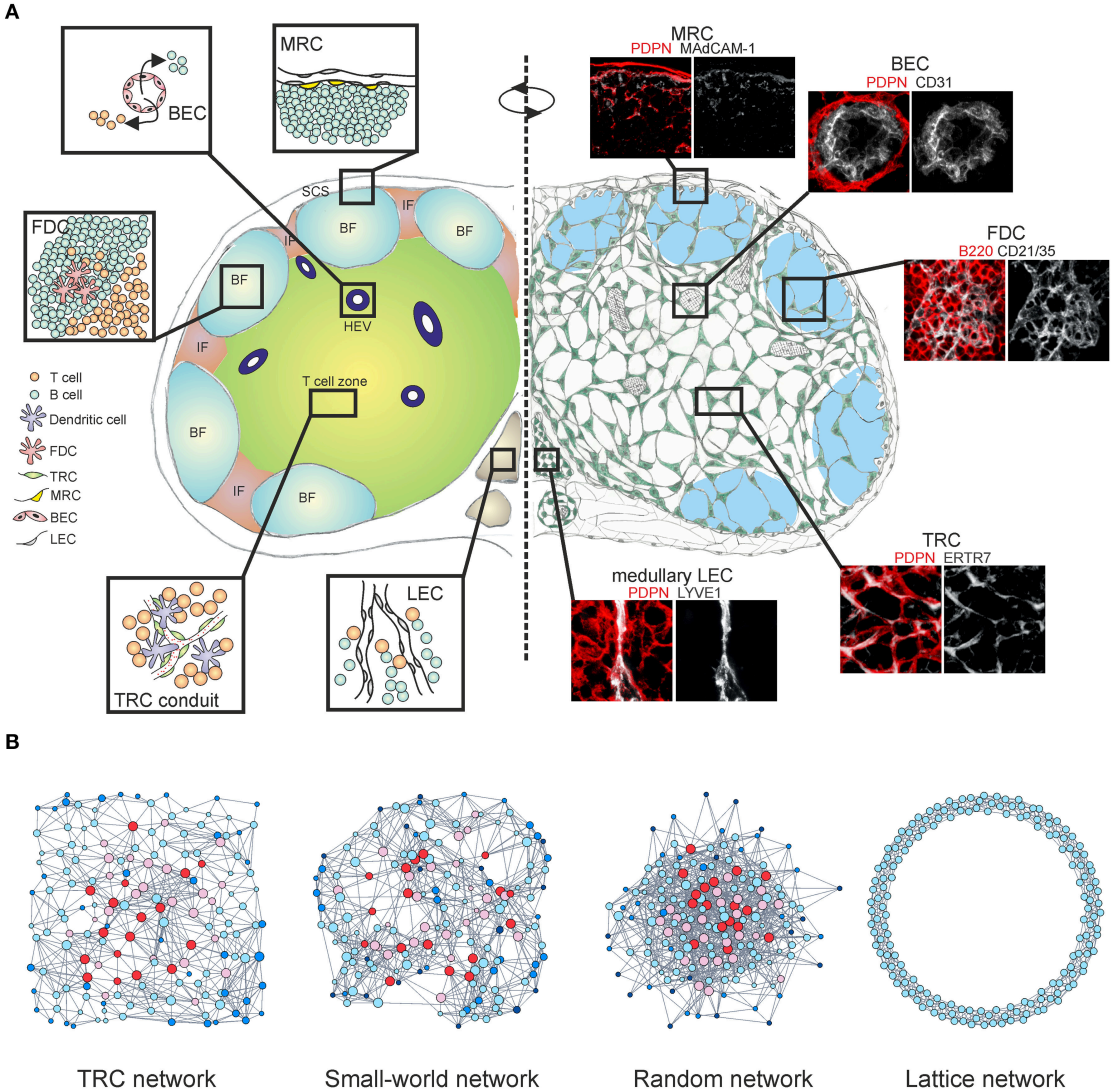
Multi-layered microarchitecture of the LN. **(A)** Schematic overview of the LN architecture and cellular organization. Zoom-in panels represent confocal microscopy images stained for indicated markers. BF, B cell follicle; IF, inter-follicular; SCS, subcapsular sinus; HEV, high endothelial venule; FDC, follicular dendritic cell; TRC, T cell zone fibroblastic reticular cell; MRC, marginal reticular cell; BEC, blood endothelial cell; LEC, lymphatic endothelial cell. **(B)** Network graphs of the TRC network and equivalent network models; Watts-Strogatz small-world network, Erdos-Renyi random network and 1D ring lattice network. Colors indicate nodes with low (blue) or high (red) betweenness centrality.

The lymphatic vascular system extends throughout the body, collecting interstitial tissue fluid through a network of initial lymphatic vessels (1). The lymph is then carried to the collecting lymphatics and distributed through lymphoid organs before returning to the venous circulation. Secondary lymphoid organs such as lymph nodes (LNs) form at bifurcation points along the lymphatic vasculature and serve as checkpoints for immune cells (2, 3). Adaptive immune responses are initiated and maintained in LNs via coordinated interactions between T cells, B cells, dendritic cells (DCs) and the LN-resident stromal cells (4–6) (Figure 1A). Traditionally, stromal cells have been described as connective tissue cells which organize the underlying LN infrastructure and cellular compartmentalization, however in recent decades their critical roles in regulation and coordination of immune responses have been established (7–9).

## Lymph node stromal cell framework

CD45^−^ non-hematopoietic stromal cells in LNs originate from mesenchymal and endothelial precursors and can be divided into four major subsets based on the expression of podoplanin (PDPN) and CD31; PDPN^−^CD31^+^ blood endothelial cells (BECs), PDPN^+^CD31^+^ lymphatic endothelial cells (LECs), PDPN^+^CD31^−^ fibroblastic reticular cells (FRCs) and PDPN^−^CD31^−^ double-negative cell fraction (10, 11). Stromal cell subsets form site-dependent niches customized for efficient interactions with immune cells, separating the LN into distinct regions (Figure 1A).

The lymph drains to the LN subcapsular sinus (SCS) through several afferent lymphatic vessels, carrying antigen, signaling molecules and immune cells. The SCS is lined with two types of LECs, the floor and ceiling LECs. It has been demonstrated that ceiling LECs express the atypical chemokine receptor CCRL1 (ACKR4) which binds CCR7 ligands CCL19 and CCL21, whilst floor LECs are devoid of its expression (12). Differential expression of CCRL1 creates chemokine gradients for DCs to migrate from the SCS to the LN parenchyma. The outer cortex of the LN under the SCS contains B cell follicles which are populated by several stromal cell subsets critical for B cell-dependent responses. B cells sense the CXCL13 gradient and migrate to the follicles in a CXCR5-dependent manner (13), where they interact with a dense network of CD21^+^CD35^+^ follicular dendritic cells (FDCs) in order to sample antigens (14, 15). A monolayer of MadCAM1^+^ marginal reticular cells (MRCs) also contributes to B cell homing by expression of CXCL13 (16) and they have also been shown to express RANKL (TNFSF11) in LNs (17). The specific expression of RANKL by MRCs was subsequently confirmed by single-cell RNA sequencing, although MadCAM1 expression could not be readily detected (18). It was previously shown that MRCs are able to proliferate and differentiate into FDCs during inflammation-induced remodeling of the B cell follicles (19), however the phenotype and function of MRCs still remain poorly understood.

Furthermore, the B cell zone-resident reticular cells alongside the expanding FDC network orchestrate germinal center formation during inflammation (20). Additional stromal cell subsets have been reported in the B cell follicle, such as the CXCL13-producing stromal cells surrounding inflamed B cell follicles (21) and a CXCL12^+^ reticular stromal subset in the dark zone of the germinal center following infection (22). Clearly, the heterogeneity of B follicle stromal cells requires further dissection in order to identify the key players in the development of humoral immunity.

The LN is a highly vascularized organ as the blood vasculature needs to deliver oxygen and nutrients to cells in the LN parenchyma. Advances in microscopy technologies have enabled 3D imaging and quantification of the topology of the entire microvascular network in LNs (23). Importantly, during inflammation the vasculature must expand in order to accommodate the increasing metabolical demand of the LN, which is achieved through proliferation of BECs and subsequent return to homeostasis by stochastic deletion of both pre-existing and newly generated blood vessels (24). The majority of lymphocytes enter the LN paracortex through specialized blood vessels called high endothelial venules (HEVs) which mediate transendothelial extravasation (25, 26). The specific roles of HEVs in lymphocyte motility and chemotaxis as opposed to capillary endothelial cells have been recently elucidated by transcriptional profiling (27).

Upon entering the LN parenchyma, T cells crawl along the FRC network searching for cognate antigen loaded on DCs (28–30). FRCs in the T cell zone (TRC) produce homeostatic chemokines CCL19 and CCL21, guiding T cells and DCs into the relevant compartments and facilitating T-DC interactions necessary for developing adaptive immunity and antiviral responses (31–33) (Figure 1A). The interaction between PDPN^+^ perivascular FRCs and the platelet-derived C-type lectin-like receptor 2 (CLEC-2) has been shown to promote VE-cadherin expression by HEVs through local sphingosine-1-phosphate (S1P) release by platelets, effectively maintaining the vascular integrity of HEVs (34). While DCs crawl on the FRC network, the interaction between PDPN^+^ FRCs and CLEC-2 on DCs induces actin cytoskeleton remodeling and promotes DC motility (35). Furthermore, the same axis permits stretching of the FRC network in order to accommodate rapid LN expansion during an immune response, whilst DC-derived lymphotoxin beta receptor (LTβR) ligands promote FRC survival by modulating PDPN expression (36–38). A recent study employing single-cell RNA sequencing has suggested the existence of nine non-endothelial stromal cell clusters within LNs, however the expression of the canonical stromal cell marker PDPN was not sufficient to distinguish the clusters on a single cell level (18).

In addition to the emerging roles of FRCs in regulation of immune responses (39), the FRC network serves a fundamental role in the formation of the LN conduit system (40, 41). The conduit system emerges as a complex branched mesh of micro-vessels from the floor of the SCS, comprising of a collagen-rich core surrounded by a microfibrillar zone and a basement membrane. A sparse network of conduits enwrapped by FDCs pervades the B cell follicles and drains the lymph through the T cell zone where it forms a dense re-entrant loop network ensheathed by the TRCs (42). The conduits rapidly transport small signaling molecules, chemokines and soluble antigens with the lymph and deliver them to the relevant stromal cells and lymphocytes (43, 44). The conduits size exclusion criterion of <70 kDa for entry of lymph-borne antigens has been recently shown to be dependent on plasmalemma vesicle associated protein (PLVAP) expression by SCS and medullary LECs (45). Ultimately the lymph is carried through the conduit system to the medullary lymphatics where it drains out of the LN through an efferent lymphatic vessel. Egress of lymphocytes occurs at the cortical and medullary sinuses through sensing of sphingosine-1-phosphate (S1P) produced by LECs (46–48).

In conclusion, stromal cells exhibit niche-specific functions and heterogeneity, indicating the complexity of their specialized interactions with immune cells. Many questions still remain open regarding their development and plasticity in homeostasis and during ongoing immune responses.

## Stromal-immune cell interaction models

T cell motility and migration patterns arise from cell-intrinsic cues such as actin polymerization and cell-extrinsic cues which include integrin-dependent adhesion, physical guidance of the microenvironment, and chemotactic gradients (49). Based on these observations it has been proposed that T cells switch between two modes of intranodal migration (50); anchorage-dependent motility mediated by engagement of LFA-1 with ICAM-1 on DCs and FRCs (31, 51), and anchorage-independent motility driven by FRC-derived chemokines and lysophosphatidic acid (LPA) (52). Moreover, a recent study demonstrated that LFA-1 and CCR7 contribute complementary and not sequentially to intranodal T cell migration. Interestingly, the authors also show that T cells migrate in a continuous sliding locomotion rather than in a caterpillar-like manner (53, 54).

Intranodal T cell motility is closely linked to search strategies employed in order to efficiently find cognate antigen loaded on DCs (55). Additionally, migration patterns are heterogeneous between T cell subsets such as CD4 and CD8, and whether they are naive, activated or memory T cells (56–59). Thus, T cells can exhibit a spectrum of search patterns, ranging from diffusive random walks analogous to Brownian motion, superdiffusive Lévy walks and subdiffusive random motion (49, 60). It has been shown in a recent report using Agent-Based Models (ABM) that naive T cells in LNs exhibit a type of superdiffusive walk which fits best as a lognormal modulated correlated random walk among the idealized computational models studied (61) (Table 1). Similarly, another ABM study demonstrated that T cell migration in inflamed LNs best fits an inverse heterogeneous correlated random walk (78).

**Table 1:** Integrative modeling frameworks for lymph node structures and processes.

**Modeling framework**	**Structures and approaches^a^**	**Processes and models^a^**	**References**
Continuous/deterministic	LN architecture 1. 2D or 3D lattice model 2. Image-based reconstruction models 3. Topology-based parameterized computational models 4. Graph theory models	Lymph flow 1. Navier-Stokes equation 2. Poiseuille equation 3. Darcy's law 4. Starling equation 5. Compartmental models Transfer of cytokines/chemokines 1. Reaction-diffusion PDEs 2. Pharmacokinetic models with ODEs or DDEs Cell population dynamics 1. ODEs 2. Compartmental models 3. Distributed parameter systems 4. Reaction-diffusion chemotaxis and haptotaxis PDEs	(23, 62–72)
Discrete/stochastic	FRC network 1. CPMs 2. Random network models Blood vascular networks 1. 3D imaging 2. Computational geometry	Cell motility 1. Physics-based models-dissipative particle dynamics based on Newton's second law of motion 2. Cellular Automata type models–CPMs or ABMs 3. Random walks models (Brownian-, Levy-, correlated walks)	(55, 59–61, 73–78)
Hybrid/multi-scale	2D (lattice-type) LN models integrated with compartmental models of the whole organism 3D anatomically resolved models of LN structures	Integrative dynamics of immune cells, humoral factors and antigens/pathogens using combination systems of ODEs, PDEs and ABM or CPM derived for single-scale processes in a computationally consistent manner	(79–84)

a *ABM, Agent-based model; CPM, Cellular-Potts model; DDE, Delay differential equation; ODE, Ordinary differential equation; PDE, Partial differential equation*.

Numerous computational T cell migration studies in LNs did not readily include the underlying reticular network structure. Furthermore, these analyses were performed using rule-derived modeling methodologies that are phenomenological in nature, rather than a biophysics-based approach (85). However, several modeling studies have simulated the TRC network with randomized connectivity and addressed its involvement in guiding T cell motion. In one study a 3D ABM approach was used to simulate infection responses in order to observe T-DC encounters and T cell differentiation in LNs under different antigen conditions (77). 3D Cellular Potts Models (CPM) offer a complementary modeling framework to simulate dynamics of T cell and DC migration alongside the TRC network. It was shown that the complex cell movement is determined by the densely packed LN environment, even though similar migratory behavior of T cells was observed whether they preferentially adhered to the TRC network or not (73). Interestingly, the study demonstrated the existence of small dynamic T cell streams within LNs, which the authors speculate occur alongside the TRC network fibers. Another study simulated migration of T cells and DCs on the TRC network and found that constraining cell movement on the TRC network does not increase the frequency of T-DC encounters compared to Brownian motion in free 3D space (75). A subsequent theoretical study confirmed in simulations that the TRC network has only a minor effect on the contact probability between T cells and DCs (76).

A question then naturally arises; do lymphocytes require the TRC network as a guiding structure for cellular movement? The answer seems evident from plethora of experimental work, corroborated by a recent reports demonstrating that deficiency in CCR7-mediated chemokine sensing and integrin LFA-1-dependent adhesion in T cells does not abrogate intranodal migration and firm attachment to the TRC network (53, 72). However, the existing theoretical models were characterized by poorly resolved sets of multi-scale control processes regulating various cell migration modes and antigen-driven functional states of immune cells. Ultimately, the theoretical framework of many modeling studies lacked the necessary quantitative data to faithfully recapitulate the stromal-immune cell interactions. In order to extend the analogy, the simulations would represent a “car with no fuel and no wheels, moving along a random road map.”

An alternative approach to examine the TRC network at a fundamental level would be to employ the theory of complex networks, also called graph theory (86, 87). Within this mathematical framework the TRC network is denoted as a series of nodes (cells) connected with edges (cell protrusions). A recent study demonstrated that the TRCs organize as a non-stochastic small-world network with highly robust topological properties, ensuring that network failure does not occur even when up to half of the network is destroyed (66, 88). Specific genetic ablation of CCL19-producing TRCs led to highly reduced numbers of hematopoietic cells in LNs and impaired intranodal migration of T cells with marked reduction in average cell speed and motility. The few T cells that did enter the LNs exhibited undirected movement around the HEVs and were not able to migrate deeper into the paracortex, despite the conduit system still being present (20, 66). The loss of FRCs and HEVs is also associated with graft-vs.-host disease after allogeneic hematopoietic stem cell transplantation and it has been recently shown that FRCs can prime alloreactive T cells through Delta-like Notch ligands (89, 90). Moreover, the TRC network is capable of fully regenerating after complete ablation and this observation is indicative of a formation of a cost-effective, optimally robust network structure that simply could not have a random configuration (91, 92) (Figure 1B). The heterogeneous topological properties of real world networks could not be explained by the random network model, thus it is likely that these networks evolved by optimizing two competitive selection criteria: high connectivity which confers efficiency of information transfer and low connection cost during formation of the network (93). Likewise, spatial embedding in many real world networks has significant confining effects on the overall topological structure by restricting the formation of long-distance connections (94, 95) (Figure 1B).

The intricate structure of LNs determines organ functionality, however the reverse also holds true; the diverse cellular interactions require a particular underlying structure to be present (96). Although it is widely accepted the TRC network serves as a “road system” for T cell and DC migration (29, 97), it remains unclear whether and to which extent dynamic cell movements are spatially constrained by the intricate network fibers (92, 98). Hence, incorporating quantitative data into integrative models may provide answers to these fundamental questions.

## Integrative lymph node models

Maintenance of chemokine gradients by stromal cells is crucial for lymphoid organ development and spatiotemporal segregation of specialized immune cell compartments (99, 100). Chemotaxis in LNs has been modeled using ABMs in order to simulate large numbers of T cells in a computationally efficient manner. By modeling T cell motion as a persistent random walk and allowing for cell crowding on a 3D lattice, a basic T cell ingress-egress model in LNs could be constructed (74). Lymphocytes must navigate efficiently within spatially heterogeneous chemokine fields that also vary over the time course of an immune response. It was shown that temporal sensing of rising chemokine concentrations is required for directional persistence of DC and neutrophil migration (101). Moreover, chemotactic-driven directional movement of DCs is steered by soluble forms of CCL19 and CCL21, whilst immobilized form of CCL21 on FRCs induces both DC motility and integrin-dependent adhesion (102).

Functional chemokine gradients of CCL19 and CCL21 have been simulated in various LN regions using a fluid flow model where the intranodal chemokine dynamics are described by ordinary (ODE) and partial differential equations (PDE) (68). Similarly, using a reaction-diffusion PDE model, highly heterogeneous distribution of IFN-α has been found, where certain LN subdomains are highly protected, whilst others are characterized by much lower levels of the cytokine (62). In a recent theoretical study, it has been demonstrated by reaction-diffusion-advection modeling that hypersensitivity in antigen recognition by immune cells can occur when chemotactic strength is higher than a predicted threshold, leading to immune system instability (69). In the case of cytokine concentration fields, it has been demonstrated that the size of cytokine niches on a single-cell level are governed by a simple mechanism dependent on cytokine diffusion and the density of consumer cells present in the niche (103).

It is important to consider another relevant aspect of LN functionality, namely lymph flow dynamics which contribute greatly to antigen, cytokine and chemokine transport. In order to gain insights into the quantitative flow parameters regulating lymph transport, a computational lymph flow model of the LN was constructed (63, 65). Interestingly, the model predicted that 90% of lymph traveled the peripheral path through the SCS and medullary sinuses. In a subsequent study the authors expanded their computational model to include intranodal CCL19 and CCL21 chemokine gradients (68). An integrative LN model with realistic 3D geometry has been recently developed in order to study lymph transport phenomena (82). The relationship between the structural LN geometry and fluid pathways has been investigated using image-based modeling of fluid flow in order to study the permeability of the LN tissue (64, 71). Furthermore, fluid flow dynamics of the blood microvasculature and the conduit system have been successfully integrated in the existing LN model (67, 70). The model predicted high robustness of the conduit system, with 60–90% elimination of conduits required to halt the lymph flux. Moreover, computational simulations of lymph flow can be expanded on larger spatial scales by modeling the entire human lymphatic system interconnected between hundreds of LNs (84).

In order to model complex biological phenomena with continuous and discrete variables, and across several spatial scales, hybrid and multi-scale modeling approaches are necessary (104–108). In recent years these models have been used to describe spatial dynamics of immune responses in LNs (79–81, 83). A summary of the integrative modeling frameworks described here and their implementation in elaborating LN processes and functions is available in Table 1. These multi-scale modeling approaches will prove invaluable in unraveling complex mechanisms of immune system control in future studies.

## Concluding remarks

Systems biology approaches have made tremendous advances in the past decade due to a high demand for bioinformatics-based computational methods necessary to describe biological systems on a global level (109). Likewise, quantitative and computational *in silico* models in immunology have become critical for understanding the emergent properties of both single cells and whole tissues (110, 111). However, the development of mathematical LN models is still confronted with technical challenges. Understanding the multi-layered compartmentalization of the LN is an important prerequisite so that the initial assumptions of the model reflect the functionality observed experimentally. To date, our knowledge of the heterogeneity of stromal cells that construct the underlying foundations of a LN is still incomplete. The directional cues und critical immunoregulatory functions of stromal cells enable the formation of specialized micro-environmental niches for immune cells within the LN, effectively facilitating immune responses (11). The described computational models largely do not take into account an additional layer of complexity, which is introduced by the fact that chemoattractant fields significantly change during inflammation and ongoing immune responses, influencing the migration and composition of immune cells. Moreover, the LN stromal compartment undergoes extensive remodeling in order to accommodate the increased LN size and proliferative demands of developing adaptive immune responses (9). Therefore, mathematical models must take into account how the spatial constraints of the LN and heterogeneous chemoattractant gradient fields affect the non-uniform distribution of immune cells, the spatiotemporal dynamics of cellular interactions and the anisotropy of non-Brownian immune cell movement patterns. To this end, quantitative data on immune cell motility metrics in homeostasis and disease/inflammatory states are critically needed for the development and calibration of biophysics-based models.

One major difficulty lies in delineating the complexity of the fundamental LN architecture and simplifying the components to a degree necessary to obtain biologically meaningful conclusions. Morphometric studies have been instrumental in describing the structural framework of distinct LN regions. However, quantitative data is still lacking for the organization of lymphatic endothelium in the medullary region, a comprehensive description of the B follicular stromal cells has not been fully elaborated and the structure of the fine-grained conduit system has not been extensively studied. Absence of detailed structural parameters represents a major caveat in data-driven systems biology approaches (112). Nevertheless, novel high-resolution imaging technologies coupled with multi-scale computational models will give us valuable insights into the inner “clockwork” of the LN.

## Author contributions

All authors contributed to writing the manuscript. Figure and data were generated by H-WC, LO, and MN, table was generated by GB.

### Conflict of interest statement

The authors declare that the research was conducted in the absence of any commercial or financial relationships that could be construed as a potential conflict of interest.
